# Fast and Environment-Friendly GC-MS Method for Eleven Organophosphorus Flame Retardants in Indoor Air, Dust, and Skin Wipes

**DOI:** 10.3390/toxics9120350

**Published:** 2021-12-11

**Authors:** Chung-Yu Chen, Yu-Hsuan Liu, Chia-Hui Chieh, Wei-Hsiang Chang

**Affiliations:** 1 Department of Occupational Safety and Health, School of Safety and Health Sciences, Chang Jung Christian University, Tainan 711, Taiwan; cyuchen@mail.cjcu.edu.tw; 2 Occupation Environment and Food Safety Research Center, Chan Jung Christian University, Tainan 711, Taiwan; 3 Department of Environmental and Occupational Health, College of Medicine, National Cheng Kung University, Tainan 704, Taiwan; liuyusyuan840921@gmail.com (Y.-H.L.); whitney860207@gmail.com (C.-H.C.); 4 Department of Food Safety/Hygiene and Risk Management, College of Medicine, National Cheng Kung University, Tainan 704, Taiwan; 5 Research Center of Environmental Trace Toxic Substances, National Cheng Kung University, Tainan 704, Taiwan

**Keywords:** organophosphorus flame retardants, indoor environment, green analytical chemistry, multimedia, GC-EI-MS

## Abstract

Organophosphorus based flame retardants (OPFRs) extensively used as alternatives to banned polybrominated diphenyl ethers and hexabromocyclododecane have been garnering interest due to the possibility that these compounds may have less significant impact on human and environmental health. Long pretreatment time, larger consumption of organic solvents, matrix interferents, and cross-contamination were found in previous studies while assessing OPFRs in indoor environments. We developed and optimized the extraction methods and simultaneous analysis of 11 OPFRs in indoor air, dust and skin wipe samples using the GC-MS approach. The proposed methods were validated using a standard addition approach, dust SRM 2585 and the real samples. Our procedures enabled the analyst to effectively limit coextracted interferences and simultaneous analytical methods of 11 target OPFRs for three matrices were achieved. The validation was performed according to standard guidelines (relative errors were identified by the analytes: −19% to 18% for indoor air, −11% to 14% for house dust, −15% to 16% for skin wipe). Good practices for quality assurance and quality control were well stated. The current high-Eco-scored methods could be categorized as “an excellent green analysis”. All analytes for the target OPFRs were detected in the real samples of indoor air, house dust and skin wipe collected from ten Taiwanese homes. Tris(2-butoxyethyl) phosphate, tris(1,3-dichloro-2-propyl)phosphate and tris(chloroisopropyl) phosphate were the most abundant OPFRs. Rapid, green and cost-effective GC-MS methods were developed and validated for the analysis of eleven OPFRs in indoor air, house dust and skin wipes.

## 1. Introduction

Currently, organophosphorus flame retardants (OPFRs), a group of flame retardants, are widely used as alternatives to polybrominated diphenyl ethers (PBDEs) and hexabromocyclododecane (HBCDD), which have been prohibited for manufacturing in Europe [1] and have been voluntarily phased out of the US market [2] due to their bioaccumulation in aquatic and terrestrial food chains and toxicity [3]. The global consumption of OPFRs has reached more than 7 million tons [4,5] and accounted for more than 55% of all OPFRs in Asian markets [6]. Due to their low cost, effectiveness and stability, OPFRs are largely adopted as plasticizer additives and flame retardants in a wide variety of consumer products and building materials, including textiles, wallpapers, foams, plastics flooring, paints and electronic equipment [7,8,9].

Humans spend 80–90% of their time indoors [10], and it is critical to characterize exposure to OPFRs in indoor environments to determine the potential sources and health risks for the general population. When OPFRs-containing products are used indoors, OPFRs can be released from these polymer or textile materials due to nonchemically bound processing and they can easily migrate into the surrounding environment through volatilization, leaching and abrasion, and direct transfer to air or dust [11,12] from consumer products. Consequently, widespread detection of OPFRs were reported in different indoor environments, including building material stores, cars, schools, homes, offices, home accessory stores, and child-care centers [9,13,14]. Nonoccupational human exposure to OPFRs mainly occurs through inhalation, ingestion and dermal contact. Among these, indoor dust ingestion and indoor air inhalation are considered the main routes of exposure in indoor environments [6,15,16]. Frequent hand-to-mouth contact and rolling-to-floor behavior might increase children’s exposure to OPFRs through the nondietary ingestion of dust adhering to their skin [17,18]. Exposure to OPFRs might result in various health effects, including endocrine disruption, reproductive dysfunction, and neuropathic damage [4,15,19,20]. Therefore, a rapid and green method to enable the simultaneous analysis (includes sample preparation and quantification) of various OPFRs in indoor air, dust, and hand wipes are required.

Several analytical approaches have been used to quantify the levels of OPFRs in environmental matrices (e.g., sediments, soil, air, surface water, drinking water, indoor air and dust) [5,21] and biological samples [22,23]. Typically, OPFRs in indoor air, dust and skin wipe samples are generally prepared by different extraction methods (e.g., Soxhlet extraction, ultrasonication, solid-liquid extraction, accelerated solvent extraction (ASE), and microwave-assisted extraction) and further cleaned up by solid-phase extraction before analysis by gas chromatography-mass spectrometry (GC-MS) [8,14,24,25,26,27,28] or liquid chromatography/mass spectrometry (LC-MS) [29,30]. The major disadvantages of the sample preparation from the above studies were the long pretreatment time, larger consumption of organic solvents, matrix interferents, and cross-contamination during complex procedures. High organic solvent consumption and laboratory plastic waste of certain analytical methods have led to environmental hazards [4,5,21]. For instance, Soxhlet extraction may take up to 24 h and often consumes 10 to 400 mL of organic solvents (e.g., acetone, toluene n-hexane, and methylene chloride) to extract OPFRs from dust or wipe samples [24]. Additional time and the amount of solvent are expended in the following cleanup steps. SPE cleanup is mostly used with florisil or silica adsorbents to remove potential interferences, resulting in the long sample preparation [14,31,32]. Therefore, more environmentally friendly analytical methods are needed to improve long-term environmental sustainability. The degree of analytical greenness is a multivariate, complex parameter that is not easily quantifiable. Continuous functions and data visualization of the approaches [33,34,35] to the guidelines of green analytical chemistry metrics [36] have been published so far.

The objectives of the study were to (i) develop and optimize a rapid and green method with effective and reliable extraction and simultaneous analysis of 11 OPFRs that most manufactured in indoor air, dust and skin wipe samples using the GC-MS approach, (ii) validate the proposed methods using standard addition approach and fully test the method performance, sensitivity and variability for indoor air, dust and skin wipe samples, and (iii) apply the validated green methods to the real samples collected from Taiwanese indoor environments to evaluate the method applicability.

## 2. Materials and Methods

### 2.1. Standards and Chemicals

Standards of 11 OPFRs include the following (Table S1): the TEP (Triethyl phosphate), TCP (Tricresyl phosphate), TIBP (Tri-iso-butyl phosphate), TNBP (Tributyl phosphate), TCEP (Tris-2-chloroethyl phosphate), TCIPP (Tris-chloroisopropyl phosphate), TDCIPP (Tris-1,3-dichloro-2-propyl phosphate), TPHP (Triphenyl phosphate), TEHP (Tris-2-ethylhexyl phosphate), TBOEP (Tris-2-butoxyethyl phosphate) and EHDPP (2-ethylhexyldiphenyl phosphate) used in this study were purchased from AccuStandard Inc. (New Haven, CT, USA). The purity of analytical standards was >96%, except for EHDPP (>91%) and TBOEP (>93%). TNBP-d27 was acquired from Cambridge Isotope Laboratories, Inc. (Tewksbury, MA, USA). Test dust (ASHRAE 52.1) was procured from Powder Technology Incorporated (PTI, Arden Hills, MN, USA). Acetone, n-hexane, dichloromethane (DCM) and toluene were purchased from Merck (Darmstadt, Germany), while ethyl acetate (EtAc) was obtained from Duksan Pure Chemicals (Ansan, South Korea). Carrier gases, helium was pure at 99.999% for GC analyses. Individual stock solutions (>1000 μg/mL) and working solutions (1 and 100 μg/mL) of native OPFRs were prepared in methanol. TNBP-d27 was used as internal standard and prepared as a standard solution (100 μg/mL) for batch analysis. All standard solutions were stored at −20 °C in amber borosilicate glass vials.

### 2.2. Sampling

The indoor air sampling approach was modified from the reported methods for analyzing air OPFRs [37,38]. The indoor air sample was gathered using XAD-2 absorbent tube (SKC 226-58, 8 × 75-mm, 140/270 mg, 20/60 mesh) with a Gilian^®^ Gilair Plus Datalogging Model Personal Sampling Pump (Sensidyne, LP, St. Petersburg, USA) flowing at 1 L/min. In the test households, duplicate air sampling tubes were used for 24 h from two sampling locations. Air sampling heads were deployed in the central area of two different rooms situated approximately at a height of 1–1.5 m from the floor, thereby (i) confirming air sampling was practiced near the breathing zone of residents and (ii) assuring children did not touch the equipment. After collection was completed, all air samples were retrieved, wrapped in aluminum foil and sealed in glass bottles. Then, the sampling tubes were taken to the laboratory maintained in a freezer and stored at −20 °C until analysis.

Dust samples from the participants’ living rooms and bedroom were vacuumed (at the standardized speed of 5 min/m^2^) into Thimble filters (28 × 100 mm, Grade 84; ADVANTEC, Toyo Roshi Kaisha, Ltd., Tokyo, Japan) using a vacuum cleaner (cleaned with acetone and methanol before sampling) equipped with a standardized aluminum-made sampling nozzle. After collection, the dust samples were packaged with a solvent-cleaned aluminum foil, sealed with parafilm, and then stored at 4 °C until dust sieving. Dust samples were homogenized, and then sieved with a stainless steel mesh sized 300 μm, to remove impurities, including stones and hairs. Fine dust samples were stored at −20 °C until analysis.

At the start of a home visit, skin wipe samples of participants were collected (wearing gloves) by wiping the full skin area of the hand, top and bottom (i.e., palm), from wrist to fingertips (including the sides of the hands and fingers) using Ghost wipes (225–2414, 10 cm × 10 cm, SKC Ltd. Blandford Forum, UK). Each participant was asked to wipe the entire hand surface at least two times using one surface of a Ghost wipe and then wiped two other times using the opposite side. All participants signed an informed consent that the ethical approval for the study protocol was obtained from the NCKUH Human Experiment and Ethics Committee. The Ghost wipe was then sealed into the amber glass container and stored at −20 °C until analysis. One field blank per sampling was also performed to confirm possible contamination during the sampling period and transportation.

### 2.3. Optimization of the Sample Preparation

**Figure 1:**
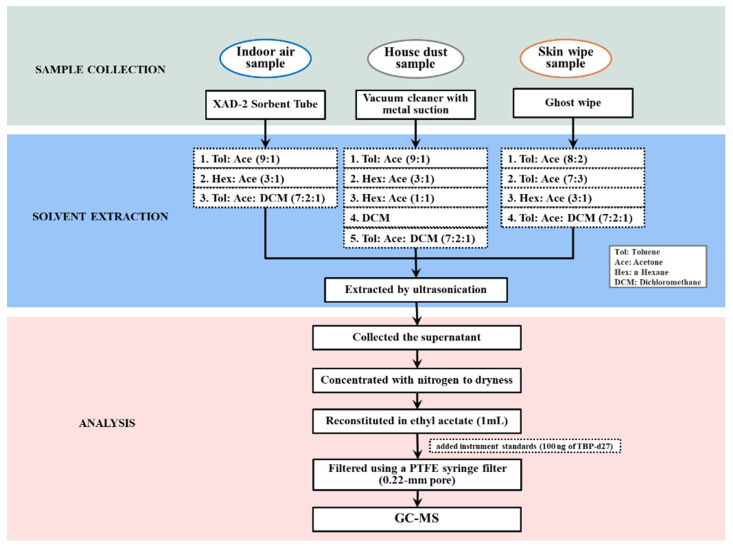
Pre-treatment tests for 11 OPFRs analysis in indoor air, house dust and skin wipe samples.

The final optimized method of extraction for the indoor air, house dust and skin wipe samples was performed based on previous studies [21,37,38]. First, solvent extraction and ultrasonication were used for sample preparation (Figure 1). Optimization experiments were performed by spiking blank XAD-2 sampling tubes, standard test dust, and blank Ghost wipes with standard solutions (0.1, 5 and 10 ppm) and testing different extraction solvents or extraction times.

The front section of the XAD-2 sorbent in an air sampling tube was placed in 8-mL glass vials and 4 mL of a solvent mixture of toluene/acetone (9:1, *v/v*) was added to the samples. The samples were given at least 15 min to equilibrate. Sample extraction was conducted by vortexing the samples for 1 min, followed by ultrasonication for 15 min. Thereafter, the extract was placed into a new cleaned glass tube, and two cycles of the procedure were conducted. The pooled extract was concentrated under sream of dried nitrogen gas, and then redissolved in 1 mL ethyl acetate. Dust samples (50 mg) that passed through a sieve mesh of 300 μm were weighed into 15-mL glass tube and then 4 mL of n-hexane/acetone (3:1, *v*/*v*) was added to the samples. The samples were equilibrated for 15 min. The extraction was carried out using ultrasonication for 30 min and then centrifuged at 2400 rpm for 10 min. Subsequently, the supernatant was placed into a test tube, and the procedure was repeated two more times. The pooled extract was concentrated to near dryness using a gentle stream of nitrogen, and redissolved in 1 mL ethyl acetate. The prepared wipe sample (cut into ~1 cm × 1 cm) was placed into 15-mL glass tube and extracted using 2 mL of a solvent mixture of n-hexane/acetone (3:1, *v*/*v*) on an ultrasonicator for 30 min. The extract was centrifuged at 3500 rpm for 10 min. Subsequently, the supernatant was placed into a new cleaned glass tube, and another two cycles of the procedure were repeated. The supernatant was redissolved in 1 mL ethyl acetate after concentration with a nitrogen dryer. Prior to analysis by GC-MS, 100 ng of TBP-d27 was added as internal standard and filtered through a 0.22-mm pore polytetrafluoroethylene (PTFE) membrane.

### 2.4. Instrumental Method

Eleven OPFR compounds were analyzed using an Agilent gas chromatography mass spectrometer (GC-7890A coupled with a GCMS 5975C, Agilent Technologies, Santa Clara, CA, USA) using a DB-5MS column (60 m × 0.25 mm i.d.; 0.25 μm film thickness), the temperature was initially set at 80 °C for 2 min, then raised to 300 °C at 15 °C min^−1^ and finally maintained for 10 min. An amount of 1.0 μL of sample volume was injected in splitless mode with an inlet temperature of 290 °C. Electron ionization (EI) was used for MS, and a temperature of 280 °C was set for the ion source and interface. MS was performed in selected ion monitoring (SIM) mode. An overview of the analytes containing detailed information for identification and quantification purposes and retention times acquired for elution on the DB-5ms capillary column is presented in Figure S1. The analytical data were processed using Mass Hunter quantitative analysis software (MassHunter WorkStation 10.2, Agilent Technologies, Santa Clara, CA, USA).

### 2.5. QA/QC and Validation Procedure

All glassware was cleaned with acetone and methanol to remove possible background contamination and baked for 1 h at 300 °C before sampling and commencement of the experiment. Characterizations for the optimized method were performed with recovery, linearity, limit of detection and quantification, precision and accuracy. Basically, recovery was estimated using the low-concentration (QCL), median-concentration (QCM) and high-concentration (QCH) spiked samples, by comparing the initial concentrations of the spiked standards. A linear curve was established by spiking eight levels of targeted OPFRs in methanol, while studying the linear relationship over a broad range (Table 1). The instrument detection limit (IDL) and method detection limit (MDL) were performed according to the guideline NIEA-PA107 published by the Taiwan Environmental Protection Administration (EPA). IDL was set as the lowest concentration of analyst that yields a separable signal (3 times) when compared to the noise peak (to comply with a reliable statistical confidence interval), while the MDL was carried out on the complete experimental procedures (in matrix) that could influence the actual levels of the target OPFRs on 2-step verification. Precision was indicated as the percentage of relative standard deviation (RSD) assayed with the variabilities among intraday and interday tests. Intraday precision was completed by analyzing three sample matrices of indoor air, dust, and skin wipe on the same day, while the interday precision was estimated over 7 days. The accuracy was determined as the difference between the measured concentration and the spiked concentration.

### 2.6. Index of Green Analytical Chemistry

The Analytical Eco-Scale [33] and AGREE Software [34] were both applied to determine the degree of greenness of analytical methods. The Analytical Eco-Scale, a semi-quantitative tool, is based on assigning penalty points to each aspect that decreases the procedure’s greenness. Points for toxic reagents (amount × hazard), waste generation, or high energetic demand are subtracted from base 100. The result of calculation is ranked on a scale, where the score: >75 represents excellent green analysis, >50 represents acceptable green analysis, <50 represents inadequate green analysis. The AGREE Software, an analytical greenness calculator, provides an easily interpretable and informative results for the 12 principles (sample treatment, sample amount, device positioning, sample preparation stages, automation/miniaturization, derivation, waste, analysis throughput, energy consumption, source of reagents, toxicity, and operator’s safety) of green analytical chemistry into scores (a unified 0−1 scale). The combination of colors that depends on the performance in each category is easy to interpret how the procedures more environmentally benign and safer to humans.

## 3. Results and Discussion

### 3.1. Optimization

#### 3.1.1. Instrument Optimization in the GC-MS System

GC-EI-MS data for the target OPFRs are listed in Table 1. The quantification was conducted by the quantifier ion, and the confirmation was performed by the qualifier ion. For three tri-alkyl phosphate esters, the ion at *m/z* 99 (corresponding to H_4_PO_4_^+^) that was attributed to undergoing three consecutive McLafferty rearrangements was frequently selected as the optimal quantifier ion using EI MS [39]. Owing to matrix effects of real samples, these intense ions of chlorinated alkyl and aryl phosphates were adopted as their quantifier ions instead of the base peak for EI–MS–SIM analysis. A typical chromatogram obtained for DB-5 of a standard solution is presented in Figure S1. The 11 OPFRs were successfully separated by the column and were detected within 44 min. Instrument conditions [37,38,40] were optimized for the injection mode, column length and monitored ions, which affect the performance of OPFRs determination. For instance, high temperatures are necessary to inject and separate OPFRs in GC systems. Most OPFR compounds are thermally labile compounds at high temperatures of approximately 300 °C [21,40,41]. Due to the difficult separation of certain OPFR components [5], the capillary column was selected based on its performance in previous studies [42,43]. Oven temperature programming was optimized to reach the best result by integrating both chromatographic efficiency and resolution of OPFR compounds.

#### 3.1.2. Sample Preparation

Ultrasonication and solvent extraction were both used for OPFRs analysis with similar or better recoveries of indoor air, dust and skin wipe samples to that in previous studies [21,37,40,44]. The extraction solvents were first optimized to enhance chromatographic separation even for the most susceptible analytes in GC-MS (Figure S1). For the air sample, we optimized the duration and volume of the solvent mixture for the extraction as follows: two extraction cycles of 1 min vortexing + 15 min ultrasonication using 4 mL of a solvent mixture of toluene/acetone (9:1, *v*/*v*) were sufficient (>95%, except TBOEP) (Figures S2 and S3). This extraction procedure was compared with two other solvent mixtures of n-hexane/acetone (3:1, *v*/*v*) and toluene/acetone/DCM (7:2:1, *v*/*v*) which gave lower recoveries and higher variability among analytes for the target OPFRs. For dust and skin wipe samples, three extraction cycles of 1 min vortexing + 30 min ultrasonication using 4 mL of a solvent mixture of n-hexane/acetone (3:1, *v*/*v*) were sufficient (mostly >80%, except TCEP in skin wipe). This extraction procedure was compared with the other solvent mixtures of toluene/acetone (9:1, 8:2, 7:3, *v*/*v*), toluene/acetone/DCM (7:2:1, *v*/*v*), n-hexane/acetone (1:1) and DCM which gave lower recoveries for all target OPFRs.

A clean-up of OPFRs extract is usually applied to minimize matrix interferences. Several sorbents/cartridges, including alumina, Oasis HLB, WAX, Envi-Carb, florisil and silica cartridges have been reported in the literature for this purpose in different environmental samples [5,21,24,37,40,45,46,47]. In this study, no further clean-up step (e.g., solid-phase extraction) was required in the sample preparation. Recoveries of the OPFRs of interest were 94.2–113%, 77.1–109% and 73.4–113% in indoor air, house dust and skin wipe samples, respectively. In comparison with published methods (based on solvent extraction, ultrasonication and clean up column) used in previous studies in the different matrices, proposed sample preparation procedures (based on solvent extraction combined with ultrasonication) could perform similar or better recoveries for target OPFRs (Table S2). This provides both cost efficiency and quickness, while the consumable operating costs utilized in our method are similar to those of the developed technologies. Thus, in addition to considering time and cost spending, the resulting impact on the environment is irresistible.

### 3.2. Method Performance

After the instrument parameters and sample preparations were optimized, the proposed method further confirmed its reliability and consistency for the characterization of the investigated OPFRs. The validation results were performed using a blank XAD sorbent tube, QC dust and blank skin wipe at three spiking levels (QCL, QCM and QCH), procedural blanks, and repeated spiked samples.

#### 3.2.1. Linearity

The linear calibration covered the entire range of concentrations in real samples of indoor air, house dust and skin wipe samples in this study, which is shown in Table 1. The linearity was evaluated on three alternating days (*n* = 8), all comprising eight levels ranging from 0.100 to 20.0 μg/mL for indoor air (0.034 to 6.87 μg/m^3^) and house dust (2.00 to 400 μg/g) samples as well as 0.050 to 10.0 μg/mL for skin wipe (2.22 to 444 μg/m^2^) samples (Table 1) and subjected to established methods. Linearity was verified for an extensive working range for all samples with regression coefficients generally higher than 0.998. The calibration curves and linearity in this study were comparable to those of other studies [37,40,46,48]. However, different calibration curves of the target OPFRs were found to not exceed 15% relative errors.

#### 3.2.2. Sensitivity

Two different detection limits were assessed, instrument sensitivity (Instrument Detection Limit, IDL) and method sensitivity (Method Detection Limit, MDL). IDL is determined as the lowest level of analyst (*n* = 7) that yields a separable peak in comparison to the noise peak (to comply with a 99% confidence interval), MDL was prepared to carry out the complete experimental procedures (in matrix) that could influence the real concentrations of the target OPFRs. The IDLs and MDLs evaluated are listed in Table 1. The estimated IDLs of the target OPFRs ranged from 0.015 to 0.030 μg/mL. The IDLs achieved for the target OPFRs were similar or lower than those found in previous works using the same detection technique [21,37,46,47,49]. The estimated MDLs of the target OPFRs ranged from 0.005 to 0.01 μg/m^3^, from 0.27 to 0.54 μg/g and from 0.08 to 0.50 μg/m^2^ in indoor air, house dust and skin wipe samples, respectively. The MDLs of different environmental matrices reported in this study provided satisfying consequences in comparison with previous works, as expected [24,37,40,46,48,50].

#### 3.2.3. Recoveries, Precision and Accuracy

Recoveries, precision and accuracy were all tested for the entire method by analysis of targeted OPFRs standards in three matrices (Table 2). Recoveries were determined by comparing the outcomes from the spiked test samples with OPFRs standard solutions (considered 100% recovery) of high (10 μg/mL), medium (5 μg/mL) and low (0.1 μg/mL) levels minus the amount found in the (unspiked) QC sample. All investigated compounds had recoveries ranging from 94.2–113%, 77.1–109% and 73.4–113% of the spiked air, dust and skin wipe samples, respectively. Recoveries (*n* = 18) of all investigated OPFRs had less than 11% RSD indicating good method precision. Intraday precision was completed by analyzing three sample matrices of indoor air, dust, and skin wipe on the same day, while the inter-day precision was estimated over 7 days. The intra- and inter-day RSDs were 2.47–9.10% and 2.37–7.23%, 3.47–10.2% and 4.13–8.83% and 3.03–10.8% and 1.60–7.33% for the spiked air, dust and skin wipe samples, respectively. Our repeated data were similar or lower than those of indoor air reported in past studies by Otake et al. (4–12%), Persson et al. (2.0–7.0%), Yoshida et al. (1.3–10.7%), Pena-Abaurrea et al. (5–27%), Haraguchi et al. (4–10%) [37,44,51]; of house dust by He et al. (1.0–27%), Van den Eede et al. (1–76%) and Persson et al. (6.0–21.0%) [21,37,40] and of skin wipe by Persson et al. (2.0–37.0%) and Xu et al. (1.0–14%) [37,48]. The accuracies of 11 OPFRs in spiked dust samples were also comparable to Wang et al. [21]. Due to the higher temperature volatilities, TEP and TCEP could not be measured precisely due to significant losses during the analytical procedure. The instrument blank and the procedural blank were performed as part of the quality control in each batch, and all blanks were below half of IDL. Studies have reported background contamination as an important issue concerning OPFRs determination [4,37,52]. We could conclude that the developed method is able to fulfill the sufficient accuracy precision and sensitivity for analyzing our targeted OPFR compounds.

### 3.3. Main Achievements

**Figure 2:**
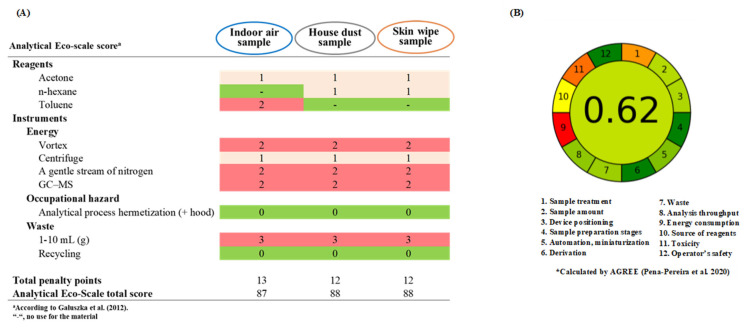
The Eco-scale score (**A**) and AGREE score (**B**) for the analytical procedures of OPFRs determinations in indoor air, house dust and skin wipe samples.

Ultrasonication, solvent extraction and centrifugation are integrated into a simple analytical method that not only reduces the extraction solvent volume but also brings out ordinary laboratory material and implicates a few easy steps. The newly proposed ultrasound-based methods easily provide the processing of 12 samples simultaneously and demand approximately 12 mL of relatively low amount of the total solvent mixtures, making use of approximately 60 min to complete an entire batch. Thus, even considering the time savings and low expenditures, the resulting environmental impact is overpowering. In contrast to the sonicated-extraction-SPE approach [24,37,40,47], the use of environmentally friendly and sustainable analytical methods presented in this study not only reduces laboratory waste, but also lessens environmental and health burdens. Assessment of the green analytical method was conducted according to the Eco-Scale proposed by Galuszka et al. [33]. The present methods can be categorized as “an excellent green analysis” for all scored above 85 in the assessments of the analytical Eco-scale (Figure 2).

### 3.4. Application of the Proposed Methods to Real Samples

#### 3.4.1. Dust SRM 2585

In order to demonstrate the efficiency and accuracy of this method, dust SRM samples were analyzed. Measured OPFRs concentrations together with reference concentrations were shown in Table S3. The differences to certified OPFRs concentrations ranged from −28.9% to 11.0%. Our result showed good agreement with most studies [21,53].

#### 3.4.2. Taiwanese Indoor Air, House Dust and Skin Wipe Samples

We employed the newly validated method to real samples of indoor air, house dust and skin wipe from ten Taiwanese homes to assess its applicability. Table 3 summarizes the OPFRs concentrations of indoor air, dust and skin wipe from ten Taiwanese homes. TEP, TCIPP, TDCIPP and TBOEP were detected in almost all samples of indoor air, dust and skin wipe, while TIBP, TNBP, TCEP, TEHP, TPHP, EHDPP and TCP had lower detection frequencies. The total levels of OPFRs ranged from <0.004–2.81 μg/m^3^ in indoor air samples, <0.200–59.7 μg/g in house dust samples and <0.100–2297 μg/m^2^ in skin wipe samples. Seven out of eleven analyzed compounds were detected, while EHDPP and TEHP were detected in one sample, TBP in two samples, and TCP was not detected in any sample. The most abundant OPFR was TDCIPP in indoor air samples, with concentrations between <0.009 and 2.81 μg/m^3^ and a mean value of 0.330 μg/m^3^; for house dust and skin wipe samples, it was TBOEP, which ranged from 11.7–78.2 μg/g and <0.10–2297 μg/m^2^ and a mean value of 31.5 μg/g and 344 μg/m^2^, respectively. High concentrations have been observed for TDCIPP (2.81 μg/m^3^), TCIPP (1.32 μg/m^3^), TPHP (1.03 μg/m^3^) and TBOEP (0.780 μg/m^3^) in indoor air samples; for TBOEP (78.2 μg/g), TDCIPP (59.7 μg/g), TCIPP (9.81 μg/g) and TEP (4.33 μg/g) in house dust samples; for TBOEP (2297 μg/m^2^), TCIPP (44.9 μg/m^2^), TDCIPP (31.8 μg/g) and TEHP (22.2 μg/g) in skin wipe samples.

As different sampling approaches have been adopted in previous studies [24,37,45,46,47,48], the comparison of OPFRs concentrations between previous studies would be limited. Our results of the OPFRs levels in indoor air denoted good agreement with OPFRs concentrations previously reported in various environments [13,25,26], but were higher than the findings of Otake et al. [51], Vykoukalova et al. [24] and Zhou et al. [14]. Compared with OPFRs concentrations of house dusts found in other countries, the OPFRs contents of house dust in this study were comparable with those in the United States [24], Canada [24], Czech Republic [24] and Netherlands [29], but were higher than those in Belgium [40], Sweden [28], China [54] and Nepal [55]. The OPFRs concentrations (especially for TBOEP) of skin wipe samples detected from Taiwanese children were approximately 3–50 times higher than those reported in American [56], Swedish [32,46], Chinese [47], Belgian [48] and Dutch [29] children. In addition, high OPFRs composition variabilities in the different matrices reported in these countries were expected.

## 4. Conclusions

Reliable, green analytical methods are largely needed to create fast, sensitive and selective measurement of emerging flame retardants in the sample matrices of indoor environments. The results of this study revealed that the established methods could be favorable alternatives for the simultaneous detection of OPFRs in the indoor air, house dust and skin wipe samples. Our optimized, green methods not only significantly enhanced the recoveries of the target OPFRs at trace levels but also notoriously reduced approximately 70–90% in time and expenditure for each sample, which provides noticeably better results compared to the previous approaches. Repeatability and good sensitivity were achieved, and the validated methods were certified to be provide good performance for all analytes and meet the suggested acceptance specification. Application of the optimum analytical method for evaluating different matrices and target analytes was successfully quantified. Generally, our results indicated that the established method could be effectively used as a simple alternative for rapid sample extraction and determination of target OPFRs in indoor air, house dust and skin wipe samples. In our study, the proposed green methods can be used for the simultaneous determination of target OPFRs for monitoring in indoor environments.

## Figures and Tables

**Table 1:** Optimized GC/MS parameters, calibration and detection limits for 11 OPFRs.

Samples/Compounds	Quantifier-Qualifier Ions	Calibration			Detection Limits (*n* = 7)	
		Linear Range	RSD (%)	*R* ^2^	IDL (μg/mL)	MDL ^a^
Indoor air (μg/m^3^)						
TEP	99, 155	0.034–6.87	5.87%	0.999	0.016	0.006
TIBP	99, 57	0.034–6.87	4.91%	0.998	0.015	0.005
TNBP	99, 151	0.034–6.87	2.69%	0.999	0.016	0.009
TCEP	63, 249	0.034–6.87	4.03%	0.999	0.019	0.007
TCIPP	99, 125	0.034–6.87	4.16%	0.999	0.030	0.010
TDCIPP	75, 77	0.034–6.87	4.24%	0.999	0.027	0.009
TPHP	99, 113	0.034–6.87	5.38%	0.998	0.023	0.008
TBOEP	57, 125	0.034–6.87	9.90%	0.998	0.028	0.010
EHDPP	251, 250	0.034–6.87	9.54%	0.999	0.019	0.007
TEHP	326, 77	0.034–6.87	9.66%	0.999	0.023	0.008
TCP	368, 367	0.034–6.87	8.50%	0.998	0.025	0.010
House dust (μg/g)						
TEP	99, 155	2.00–400	5.87%	0.999	0.016	0.325
TIBP	99, 57	2.00–400	4.91%	0.998	0.015	0.301
TNBP	99, 151	2.00–400	2.69%	0.999	0.016	0.313
TCEP	63, 249	2.00–400	4.03%	0.999	0.019	0.269
TCIPP	99, 125	2.00–400	4.16%	0.999	0.030	0.539
TDCIPP	75, 77	2.00–400	4.24%	0.999	0.027	0.540
TPHP	99, 113	2.00–400	5.38%	0.998	0.023	0.34
TBOEP	57, 125	2.00–400	9.90%	0.998	0.028	0.502
EHDPP	251, 250	2.00–400	9.54%	0.999	0.019	0.342
TEHP	326, 77	2.00–400	9.66%	0.999	0.023	0.310
TCP	368, 367	2.00–400	8.50%	0.998	0.025	0.508
Skin wipe (μg/m^2^)						
TEP	99, 155	2.22–444	4.64%	0.999	0.016	0.111
TIBP	99, 57	2.22–444	11.0%	0.999	0.015	0.076
TNBP	99, 151	2.22–444	13.1%	0.998	0.016	0.093
TCEP	63, 249	2.22–444	13.3%	0.998	0.019	0.351
TCIPP	99, 125	2.22–444	12.3%	0.998	0.030	0.351
TDCIPP	75, 77	2.22–444	13.0%	0.999	0.027	0.351
TPHP	99, 113	2.22–444	14.4%	0.999	0.023	0.133
TBOEP	57, 125	2.22–444	13.8%	0.999	0.028	0.498
EHDPP	251, 250	2.22–444	14.2%	0.999	0.019	0.098
TEHP	326, 77	2.22–444	13.4%	0.999	0.023	0.413
TCP	368, 367	2.22–444	11.8%	0.998	0.025	0.316

RSD: relative standard deviation. ^a^ Statistical pooled 7 times the standard deviation divided by the weight/volume/area of sample matrices (1.45 m^3^ for air, 0.05 g for dust and m^2^ for skin). Air sample: μg/m^3^, Dust sample: μg/g, Skin wipe sample: μg/m^2^.

**Table 2:** Summary of method performance results in indoor air, house dust and skin wipe.

Selected OPFRs	Rec (%) (*n* = 18)	Intra-Day (*n* = 9)						Inter-Day (*n* = 9)					
		0.1 ppm		5 ppm		10 ppm		0.1 ppm		5 ppm		10 ppm	
		RSD (%)	Accu (%)	RSD (%)	Accu (%)	RSD (%)	Accu (%)	RSD (%)	Accu (%)	RSD (%)	Accu (%)	RSD (%)	Accu (%)
Indoor air (μg/m^3^)													
TEP	105	1.80	1.98	5.30	4.28	7.90	4.69	2.20	3.95	1.10	6.03	3.80	−10.9
TIBP	113	7.90	6.96	9.20	5.19	10.2	7.95	6.00	6.91	2.70	5.50	3.30	4.85
TNBP	110	3.40	3.91	1.10	2.79	4.50	4.90	4.40	6.62	2.50	3.51	0.700	2.99
TCEP	100	4.10	−5.11	6.70	5.96	8.40	5.20	6.10	13.2	3.70	5.65	3.10	5.88
TCIPP	104	9.30	6.39	6.30	7.58	2.00	−4.00	3.50	9.52	4.40	7.89	3.80	−6.74
TDCIPP	110	3.70	2.54	1.90	−3.51	5.90	4.88	1.40	4.74	2.50	8.67	3.60	4.70
TPHP	94.2	3.10	4.02	6.40	−7.53	9.10	−8.13	8.20	6.93	1.10	18.9	3.30	−11.9
TBOEP	98.3	2.90	2.79	3.70	−8.39	9.60	−7.18	5.10	11.3	14.4	12.6	2.20	−19.5
EHDPP	95.9	2.40	5.61	5.70	5.52	5.00	−7.31	8.90	7.95	3.20	8.42	5.00	9.82
TEHP	98.1	1.30	−5.60	9.60	5.58	7.70	4.79	4.30	10.1	7.20	9.66	3.70	10.0
TCP	95.0	2.30	−4.01	2.20	−5.83	2.90	−4.25	5.70	4.15	3.90	6.68	2.40	−4.81
House dust (μg/g)													
TEP	79.4	6.70	−2.25	4.10	−5.06	3.20	−2.69	11.8	−9.65	6.00	−9.01	2.20	−2.90
TIBP	94.6	7.30	6.97	4.30	3.89	9.10	5.79	6.60	−7.38	3.40	−4.04	2.40	−10.9
TNBP	94.6	10.1	−3.36	4.50	3.34	11.6	−11.5	10.0	−8.22	2.10	1.78	11.5	10.2
TCEP	77.1	6.90	−6.94	3.50	−5.82	9.80	−8.70	7.00	−6.14	8.10	−6.53	7.60	−4.80
TCIPP	109	4.70	7.30	6.00	3.15	12.4	10.8	9.90	7.64	0.30	6.15	9.20	9.80
TDCIPP	94.8	5.00	6.45	3.70	−4.28	7.10	−8.90	7.90	7.44	4.90	11.6	10.7	13.4
TPHP	106	11.6	4.58	9.10	1.72	10.0	11.2	13.5	6.58	2.80	7.63	10.0	3.25
TBOEP	88.0	0.700	−3.94	1.10	−4.45	8.60	−9.79	8.40	4.67	2.40	−4.86	13.9	3.82
EHDPP	103	9.60	7.75	3.40	5.94	2.20	−9.98	11.4	7.69	1.50	11.7	12.1	9.20
TEHP	102	9.20	7.10	7.40	2.43	7.90	7.16	13.3	9.07	1.50	−2.74	11.7	14.4
TCP	105	7.80	8.04	7.40	4.32	10.6	7.40	8.30	7.51	1.30	10.1	4.20	7.67
Skin wipe (μg/m^2^)													
TEP	74.8	8.50	−4.55	2.30	−1.82	8.20	−4.91	8.20	−3.82	1.90	−2.58	1.70	−1.59
TIBP	106	13.0	−6.84	1.70	−1.67	6.60	5.54	9.40	3.90	5.50	2.90	7.10	11.8
TNBP	104	6.20	−7.41	6.50	−1.06	6.00	5.64	5.10	12.1	1.50	3.74	8.80	12.8
TCEP	73.4	6.80	−2.89	3.40	−2.66	3.90	−3.99	1.10	−2.75	1.90	−2.64	1.80	−5.58
TCIPP	113	14.0	−3.29	17.3	−9.33	1.30	8.40	7.00	5.73	3.10	10.7	5.30	8.33
TDCIPP	104	5.60	−5.17	3.80	−2.49	0.600	−4.43	3.30	5.88	1.20	14.7	5.20	16.1
TPHP	113	2.70	−6.15	3.70	−2.26	3.20	2.85	3.00	−11.1	0.800	2.15	5.10	3.59
TBOEP	90.2	10.5	12.4	7.10	1.65	4.10	5.67	5.90	0.62	1.10	14.5	12.4	12.6
EHDPP	98.8	4.00	−1.93	3.20	−4.58	1.90	−1.08	1.00	−6.68	6.00	−5.35	1.90	1.43
TEHP	87.0	9.30	−8.08	0.10	−1.30	2.90	−7.91	6.60	−9.14	2.50	4.14	8.10	9.50
TCP	99.0	2.30	−8.07	8.80	−5.58	10.3	2.70	3.10	−15.1	6.20	10.8	5.80	7.29

**Table 3:** Analysis results of real indoor air, house dust and skin wipe samples from children and their households.

	Homes	TEP	TIBP	TNBP	TCEP	TCIPP	TDCIPP	TBOEP	TEHP	TPHP	EHDPP	TCP
Indoor air (μg/m^3^)	H 1	<0.006	0.27	<0.009	<0.007	0.360	<0.009	<0.01	<0.004	<0.004	<0.004	<0.004
	H 2	0.300	0.10	<0.009	<0.007	0.370	<0.009	<0.01	<0.004	<0.004	<0.004	0.010
	H 3	0.170	<0.005	<0.009	<0.007	<0.01	<0.009	<0.01	<0.004	<0.004	<0.004	<0.004
	H 4	<0.006	<0.005	<0.009	<0.007	0.530	0.070	0.22	<0.004	<0.004	<0.004	<0.004
	H 5	<0.006	<0.005	<0.009	0.04	0.300	0.070	0.25	<0.004	<0.004	<0.004	<0.004
	H 6	<0.006	<0.005	<0.009	0.03	0.460	0.260	0.78	<0.004	0.25	<0.004	<0.004
	H 7	0.060	<0.005	0.040	0.08	1.10	2.81	0.74	<0.004	1.03	<0.004	<0.004
	H 8	0.040	<0.005	<0.009	<0.007	1.04	<0.009	0.08	<0.004	<0.004	<0.004	<0.004
	H 9	<0.006	<0.005	<0.009	<0.007	0.450	<0.009	0.05	<0.004	<0.004	<0.004	<0.004
	H 10	0.110	<0.005	0.060	<0.007	1.32	<0.009	0.08	<0.004	<0.004	<0.004	<0.004
House dust (μg/g)	H 1	2.04	<0.300	<0.310	<0.270	2.45	<0.54	11.7	<0.310	<0.340	<0.340	<0.510
	H 2	2.20	<0.300	<0.310	<0.270	2.37	3.58	39.1	<0.310	2.80	<0.340	<0.510
	H 3	3.17	<0.300	<0.310	<0.270	<0.54	59.7	62.1	<0.310	<0.340	<0.340	<0.510
	H 4	3.20	<0.300	<0.310	<0.270	9.81	2.71	78.2	<0.310	<0.340	<0.340	<0.510
	H 5	2.50	<0.300	<0.310	<0.270	3.28	2.83	33.5	<0.310	<0.340	<0.340	<0.510
	H 6	<0.330	<0.300	<0.310	<0.270	<0.54	<0.54	12.8	<0.310	<0.340	<0.340	<0.510
	H 7	<0.330	<0.300	<0.310	<0.270	<0.54	9.87	14.5	<0.310	<0.340	<0.340	<0.510
	H 8	2.15	<0.300	<0.310	<0.270	2.25	<0.54	31.7	<0.310	<0.340	<0.340	<0.510
	H 9	<0.330	<0.300	<0.310	<0.270	<0.54	<0.54	18.4	<0.310	<0.340	<0.340	<0.510
	H 10	4.33	<0.300	<0.310	<0.270	9.38	<0.54	13.4	<0.310	<0.340	<0.340	<0.510
Skin wipe (μg/m^2^)	H 1	4.90	<0.080	<0.090	<0.350	<0.35	<0.35	<0.500	<0.410	<0.130	<0.100	<0.320
	H 2	3.68	<0.080	<0.090	<0.350	<0.35	<0.35	<0.500	<0.410	<0.130	<0.100	<0.320
	H 3	<0.110	<0.080	<0.090	<0.350	4.39	<0.35	2297	22.2	<0.130	<0.100	<0.320
	H 4	<0.110	<0.080	<0.090	<0.350	25.0	31.8	82.2	<0.410	<0.130	<0.100	<0.320
	H 5	<0.110	<0.080	<0.090	<0.350	8.89	<0.35	10.2	<0.410	<0.130	<0.100	<0.320
	H 6	<0.110	<0.080	<0.090	<0.350	44.9	<0.35	304	<0.410	3.56	<0.100	<0.320
	H 7	<0.110	<0.080	<0.090	<0.350	25.8	13.4	128	<0.410	<0.130	<0.100	<0.320
	H 8	<0.110	<0.080	<0.090	<0.350	18.7	2.46	231	<0.410	<0.130	<0.100	<0.320
	H 9	<0.110	<0.080	<0.090	<0.350	16.4	32.0	165	<0.410	3.11	<0.100	<0.320
	H 10	<0.110	<0.080	<0.090	8.02	18.2	3.64	217	<0.410	6.22	8.89	<0.320

## Data Availability

The data are not publicly available due to protection of subjects’ privacy and confidentiality. The data presented in this study are available on request from the corresponding author.

## Supplementary Materials

The following are available online at https://www.mdpi.com/article/10.3390/toxics9120350/s1, Figure S1: Selected Ion Monitor chromatogram of a standard solution of eleven OPFRs (5 μg/mL), Figure S2: Spike (5.0 µg/mL) recovery rate of different matrix in different solvents and proportion, Figure S3: The spike (5.0 µg/mL) recovery rate of different matrix in different extraction times, Table S1: Summary of the names, practical abbreviations (Abbr.), CAS Number, molecular structures, molecular formulas, molecular weight, Solubility, Vapor pressure, log Koa, log Kow and Bioconcentration Factor (BCFs) of the analyzed OPFRs, Table S2: Comparisons of analytical methods and recovery to previous studies, Table S3: Measured and reference concentrations (μg/g) of selected OPFRs in dust SRM.
