# Supplementary material for: Fast and Environment-Friendly GC-MS Method for Eleven Organophosphorus Flame Retardants in Indoor Air, Dust, and Skin Wipes
Source: Toxics. 2021 Dec 11;9(12):350. doi: 10.3390/toxics9120350 (PMC8707019; doi:10.3390/toxics9120350)
Supplement: Supplementary file 1 [file toxics-09-00350-s001.zip › toxics-1474606-supplementary.pdf]

toxics

Supplementary Materials: Fast and Environment-Friendly GC-MS Method for Eleven Organophosphorus Flame Retardants in Indoor Air, Dust, and Skin Wipes

Chung-Yu Chen, Yu-Hsuan Liu, Chia-Hui Chieh and Wei-Hsiang Chang

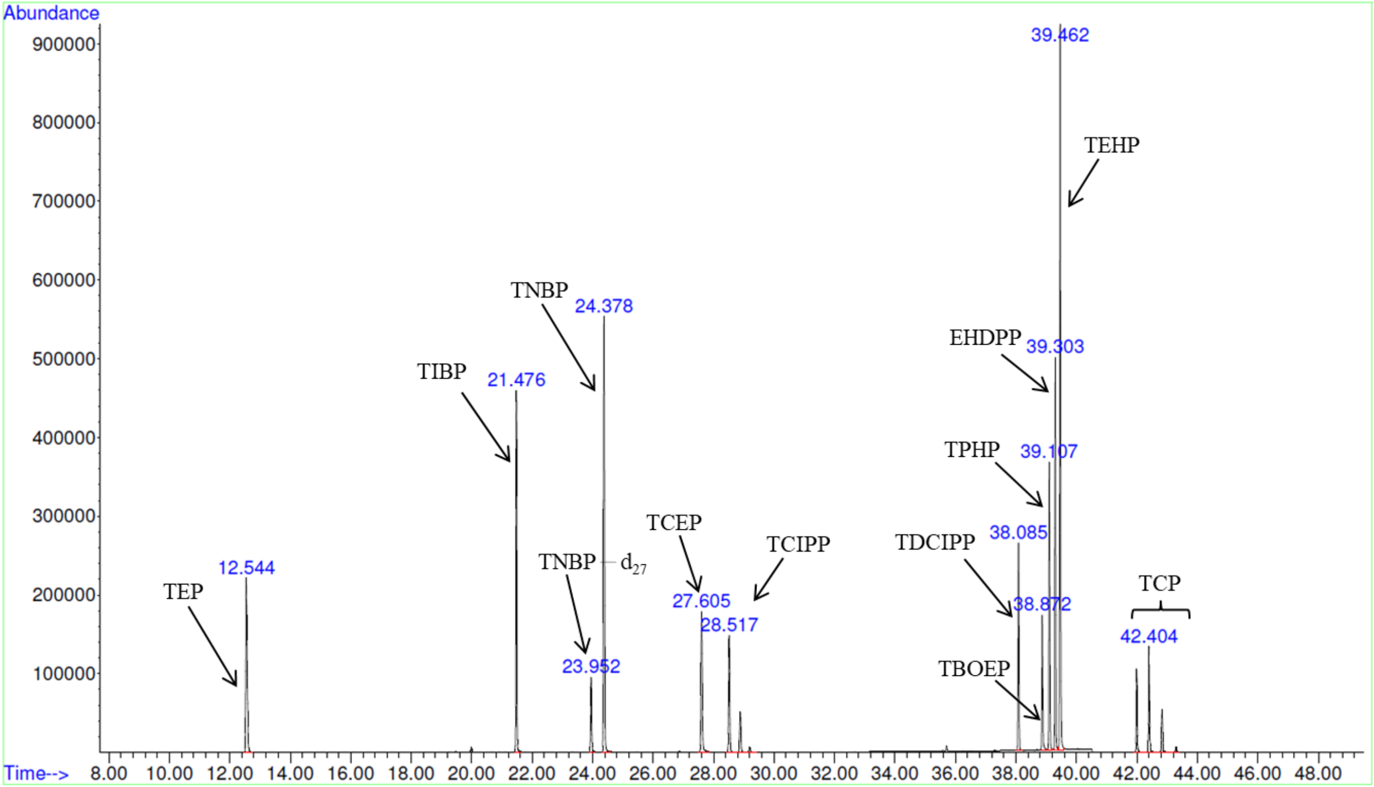

Figure S1. Selected Ion Monitor chromatogram of a standard solution of eleven OPFRs (5 µg/mL).

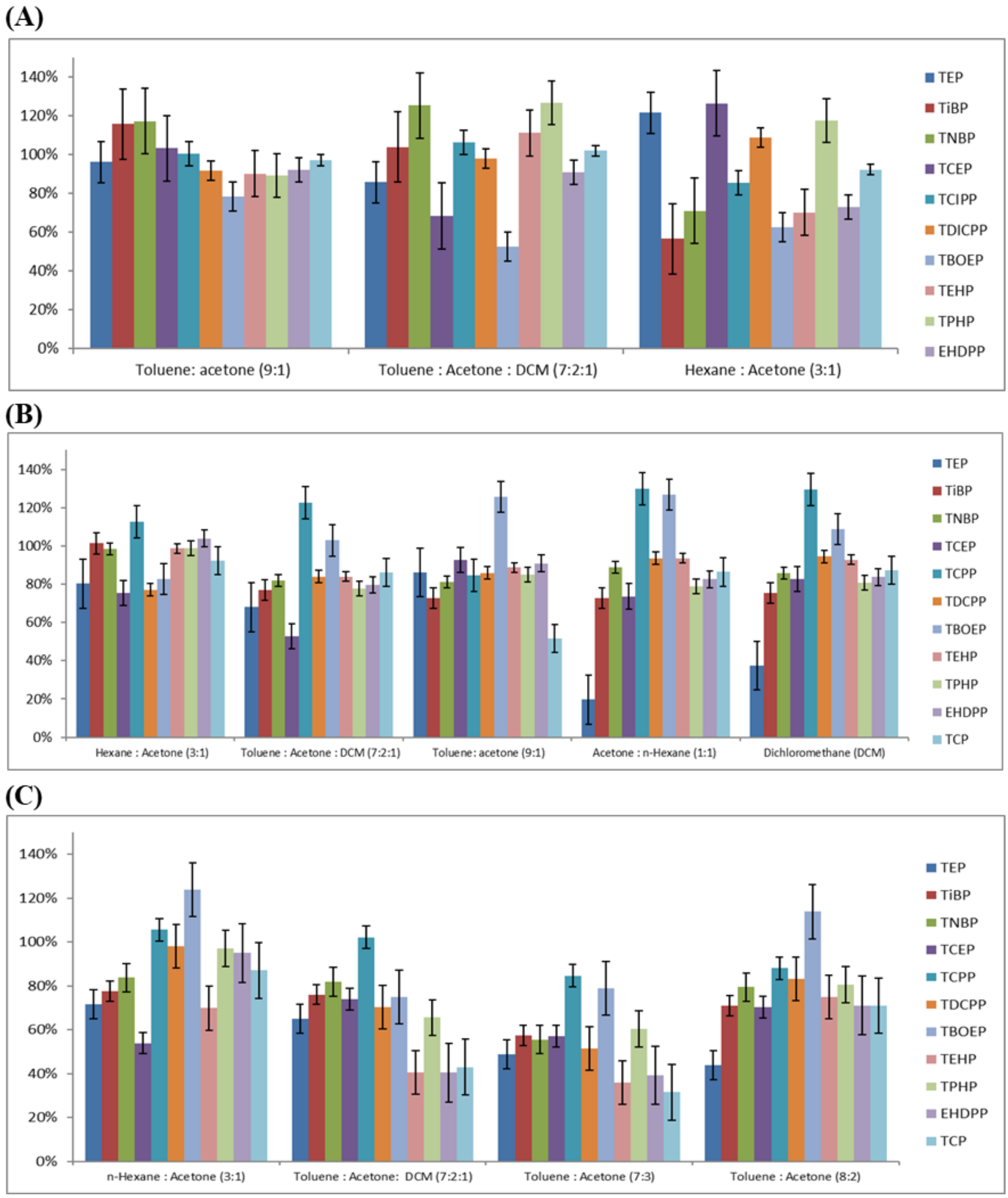

Figure S2. Spike (5.0 μg/ml) recovery rate of different matrix in different solvents and proportion **(A)** Indoor air samples, **(B)** House dust samples and **(C)** Dermal wipe samples.

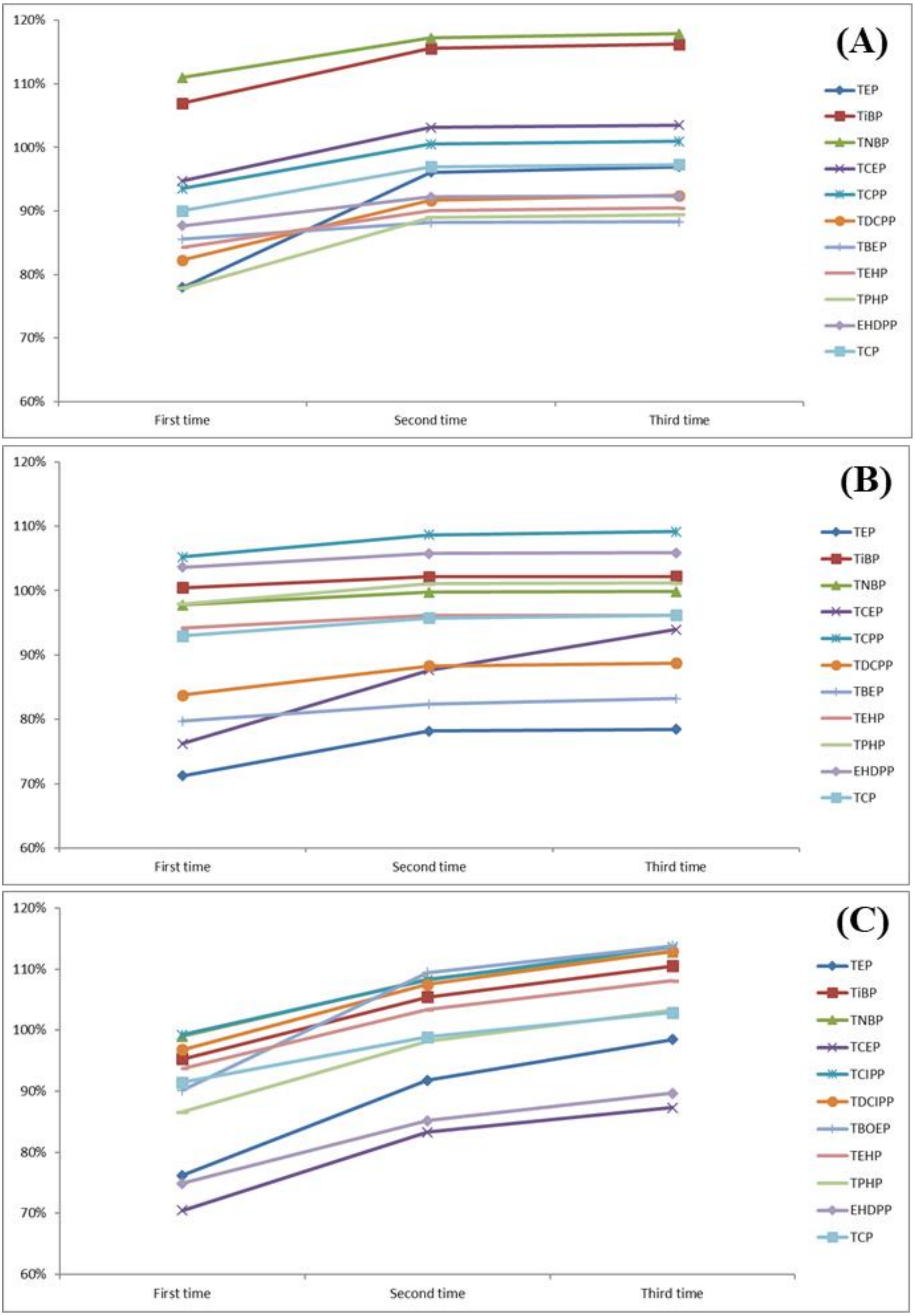

Figure S3. The spike (5.0 μg/ml) recovery rate of different matrix in different extraction times **(A)** Indoor air samples, **(B)** House dust samples and **(C)** Dermal wipe samples..

Table S1. Summary of the names, practical abbreviations(Abbr.), CAS Number, molecular structures, molecular formulas, molecular weight, Solubility, Vapor pressure, log K_{oa}, log K_{ow} and Bioconcentration Factor(BCFs) of the analyzed OPFRs

**Chemical species**	**Compound**	**Abbr.**	**CAS no.**	**Molecular structure**	**Molecular Formula**	**Molecular weight**	**Solubility (mg/L, 25℃)**	**Vapor pressure (mmHg, 25℃)**
Non-Cl alkyl phosphate	Triethyl phosphate	TEP	78-40-0	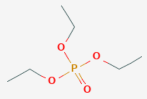	$C_6H_{15}O_4P$	182.15	11.5	3.93×10^{-1}
	Tributyl phosphate	TNBP	126-73-8	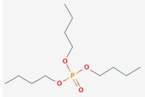	$C_{12}H_{27}O_4P$	266.31	280	1.13×10^{-3}
	Tri-iso-butyl phosphate	TiBP	126-71-6	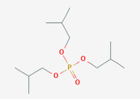	$C_{12}H_{27}O_4P$	266.31	475.6	1.29×10^{-2}
	Tris(2-ethylhexyl) phosphate	TEHP	78-42-2	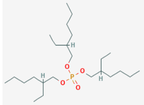	$C_{24}H_{51}O_4P$	434.63	0.6	8.25×10^{-8}
Aryl phosphate	Triphenyl phosphate	TPHP	115-86-6	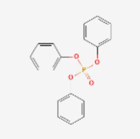	$C_{18}H_{15}O_4P$	326.28	1.9	1.12×10^{-5}
	Tricresyl phosphate	TCP	1330-78-5	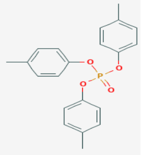	$C_{21}H_{21}O_4P$	368.37	0.36	1.8×10^{-7}
Cl alkyl phosphate	Tris(2-chloroethyl) phosphate	TCEP	115-96-8	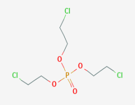	$C_6H_{12}Cl_3O_4P$	285.48	7000	6.13×10^{-2}
	Tris(chloroisopropyl) phosphate	TCIPP	13674-84-5	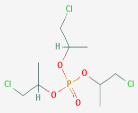	$C_9H_{18}Cl_3O_4P$	327.56	51.9	5.64×10^{-5}
	Tris(1,3-dichloro-2-propyl)phosphate	TDCIPP	13674-87-8	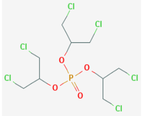	$C_9H_{15}Cl_6O_4P$	430.89	7	2.98×10^{-7}

Other phosphate	Tris(2-butoxyethyl) phosphate	TBOEP	78-51-3	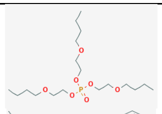	$C_{18}H_{39}O_7P$	398.47	1100	1.23×10^{-6}
	2-ethylhexyldiphenyl phosphate	EHDPP	109925-03-3	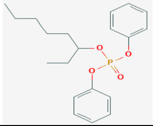	$C_{20}H_{27}O_4P$	362.41	1.9	5×10^{-5}

Abbr: abbreviation; CAS no: chemical abstract service number; log Kow: octanol-water partition coefficient; log Koa: octanol-air partition coefficient BCF: bioaccumulation factor. The data are compiled form (Q Wang et al. 2017), Hazardous Substances Data Bank (HSDB) of TOXNET, accessed at 16 June, 2020.

Table S2. Comparisons of analytical methods and recovery to previous studies.

Sampling matrix	Solvent	Extraction	Clean-up	Instrumental analysis	Recovery (%)	Reference
Indoor air samples						
XAD-2 absorbents	toluene: acetone (9:1, *v/v*)	Ultra-sonication	-	GC-MS	94.2–113	Present study
XAD-2 absorbents	n-hexane/acetone (3:1, *v/v*)	Ultra-sonication	Florisil column	GC-MS	75–172	1
polyurethane foam	Dichloromethane (DCM)	Soxhlet extraction	Silica column	GC-MS, GC-MS/MS	70–120	2
House dust samples						
Socks (ASHRAE#1 test dust)	n-hexane/acetone (3:1, *v/v*)	Ultra-sonication	-	GC-MS	77.1–109	Present study
Indoor dust	dichloromethane	Ultra-sonication	Florisil column	GC-MS	21–127	3
Cellulose filters (SRM 2582)	n-hexane/acetone (3:1, *v/v*)	Ultra-sonication	Florisil column	GC-MS	57–150	1
Socks	n-hexane/acetone (1:1, *v/v*)	Ultra-sonication	Silica column	GC-MS/MS	70–120	2
Nylon sampling sock	n-hexane/acetone (3/1, *v/v*)	Ultra-sonication	Florisil column	GC–MS	49–130	4
Forensic filters (SRM2585)	n-hexane/acetone (3:1, *v/v*)	Ultra-sonication	Florisil column	GC-EI/MS	43–111 (TBOEP 172 - 268)	5
Skin wipe samples						
Ghost wipe	n-hexane/acetone (3:1, *v/v*)	Ultra-sonication	-	GC-MS	73.4–113	Present study
Gauze Pads in isopropanol	n-hexane/acetone (3:1, *v/v*)	Ultra-sonication	Silica column	GC-MS	70–130%	6
Gauze soaked in isopropanol	n-hexane/acetone (1:1, *v/v*)	Ultra-sonication	Silica column	GC-MS	41–134	7
Kleenex tissue	n-hexane/acetone (3:1, *v/v*)	Ultra-sonication	Florisil column	GC-MS	74–100	1
Gauze soaked in isopropanol	n-hexane/acetone (3/1, *v/v*)	Ultra-sonication	Florisil column	GC–MS	49–130	4

Table S3. Measured and reference concentrations (μg/g) of selected OPFRs in dust SRM.

	Present study (n = 5)		Certified values[a]		Percent error
	Mean	SD	Mean	SD	(%)
TNBP	196	2.19	276	14	−28.9
TCEP	993	5.47	925	149	7.16
TCIPP	1366	110	1220	350	11.0
TPHP	1103	52.5	1190	130	−7.62

[a]According to Gałuszka et al. (2012). "-", no use for the material

References

1. Persson, J.; Wang, T.; Hagberg, J. Organophosphate flame retardants and plasticizers in indoor dust, air and window wipes in newly built low-energy preschools. *Sci. Total Environ.* **2018**, *628–629*, 159–168, doi:10.1016/j.scitotenv.2018.02.053.
2. Vykoukalova, M.; Venier, M.; Vojta, S.; Melymuk, L.; Becanova, J.; Romanak, K.; Prokes, R.; Okeme, J.O.; Saini, A.; Diamond, M.L.; et al. Organophosphate esters flame retardants in the indoor environment. *Environ. Int.* **2017**, *106*, 97–104, doi:10.1016/j.envint.2017.05.020.
3. Van den Eede, N.; Dirtu, A.C.; Neels, H.; Covaci, A. Analytical developments and preliminary assessment of human exposure to organophosphate flame retardants from indoor dust. *Environ. Int.* **2011**, *37*, 454–461, doi:10.1016/j.envint.2010.11.010.
4. Liu, X.; Yu, G.; Cao, Z.; Wang, B.; Huang, J.; Deng, S.; Wang, Y. Occurrence of organophosphorus flame retardants on skin wipes: Insight into human exposure from dermal absorption. *Environ. Int.* **2017**, *98*, 113–119, doi:10.1016/j.envint.2016.10.021.
5. Cequier, E.; Ionas, A.C.; Covaci, A.; Marce, R.M.; Becher, G.; Thomsen, C. Occurrence of a broad range of legacy and emerging flame retardants in indoor environments in Norway. *Environ. Sci. Technol.* **2014**, *48*, 6827–6835, doi:10.1021/es500516u.
6. Xu, F.; Giovanoulis, G.; van Waes, S.; Padilla-Sanchez, J.A.; Papadopoulou, E.; Magner, J.; Haug, L.S.; Neels, H.; Covaci, A. Comprehensive study of human external exposure to organophosphate flame retardants via air, dust, and hand wipes: The Importance of sampling and assessment strategy. *Environ. Sci. Technol.* **2016**, *50*, 7752–7760, doi:10.1021/acs.est.6b00246.
7. Larsson, K.; de Wit, C.A.; Sellstrom, U.; Sahlstrom, L.; Lindh, C.H.; Berglund, M. Brominated flame retardants and organophosphate esters in preschool dust and children's hand wipes. *Environ. Sci. Technol.* **2018**, *52*, 4878–4888, doi:10.1021/acs.est.8b00184.
